# How Breathing Exercises Influence on Respiratory Muscles and Quality of Life among Patients with COPD? A Systematic Review and Meta-Analysis

**DOI:** 10.1155/2021/1904231

**Published:** 2021-01-29

**Authors:** Ruisheng Yun, Yiwen Bai, Yan Lu, Xubo Wu, Shin-Da Lee

**Affiliations:** ^1^School of Rehabilitation Science, Shanghai University of Traditional Chinese Medicine, Shanghai 201203, China; ^2^Department of Rehabilitation Medicine, Seventh People's Hospital of Shanghai University of Traditional Chinese Medicine, Shanghai 200137, China; ^3^Department of Physical Therapy, Asia University, Taichung 41354, Taiwan; ^4^Department of Physical Therapy, Graduate Institute of Rehabilitation Science, China Medical University, Taichung 40402, Taiwan

## Abstract

**Aim:**

This systematic review aimed to investigate the effect of different breathing exercises on respiratory muscle function, 6-minute walk test (6MWT), and quality of life (QoL) in patients with chronic obstructive pulmonary disease (COPD).

**Methods:**

We searched online databases including PubMed, Embase, Web of Science, Cochrane Library, and Ovid for randomized controlled trials that assessed the efficacy of breathing exercises on patients with COPD. Patient outcome parameters included changes in respiratory muscle function, 6MWT, and QoL. The Cochrane Collaboration tool was used to assess the risk of bias for each included study. Subgroup analyses concerning different interventions and outcome measurements were conducted as necessary. PROSPERO registration number is CRD42018118367.

**Results:**

A total of 17 studies were included for final analysis. Meta-analysis based on the relevant studies showed that breathing exercises had a significant total effect on pulmonary function PImax (mean difference (MD) = 8.65, 95% confidence interval (CI) 3.13–14.16, *P*=0.002), as well as 6MWT (MD = 27.70, 95% CI 5.45–49.94, *P*=0.01) in patients with COPD.

**Conclusions:**

This systematic review summarized the use of breathing exercises for treating patients with COPD. Breathing exercises were found to be an effective tool for treating patients with COPD by improving inspiratory muscle strength and 6MWT. However, breathing exercises showed no significant improvements on the QoL of patients with COPD.

## 1. Introduction

Chronic obstructive pulmonary disease (COPD) is a chronic and progressive disease that causes irreversible airflow obstruction, and is often characterized by dyspnea, coughing, sputum production, wheezing, and chest tightness [1]. Patients with COPD usually have physiological abnormalities that are mainly associated with abnormal alterations in the pattern of ventilatory muscle recruitment. This occurs due to changes in the rib cage geometry caused by lung hyperinflation, which alters the length-tension curve of the diaphragm muscle [2]. Respiratory muscle dysfunction is frequently observed in patients with COPD [3, 4]. Weakening of the respiratory muscle in COPD patients often leads to hypercapnia, dyspnea, and decreased exercise capacity [5]. Additionally, the compensatory increase in the demand of the respiratory muscles may further exacerbate respiratory muscle dysfunction in patients with COPD [6]. Therefore, the implementation of breathing exercises that can enhance respiratory muscle function and potentially reduce the severity of symptoms in patients with COPD is critical.

Breathing exercise is defined as any breathing technique that can allow deeper inspiration or expiration, or otherwise alter the rate, pattern, or rhythm of respiration, and common examples of such exercises include inspiratory muscle training (IMT), expiratory muscle training (EMT), diaphragmatic breathing (DB), Liuzijue, and combined training exercises. These exercises can be performed with or without external devices, both during exercise or at rest [7], and belong to the category of analytical, diagnostic, and therapeutic techniques for the treatment of COPD. IMT is a breathing exercise that can delay the deterioration of lung function via increasing inspiratory muscle strength and endurance, thereby relieving dyspnea and improving the patient's quality of life (QoL) [8, 9]. Similarly, EMT helps in maintaining respiratory function and cough function [10]. Other breathing exercises, such as Liuzijue (a traditional Chinese breathing exercise that combines the respiratory patterns of abdominal breathing and pursed-lip breathing), have been shown to be effective and feasible for use in elderly patients with moderate-to-severe COPD [11]. DB, also commonly known as breathing control or abdominal breathing, is known to improve tidal volume, oxygen saturation, ventilation, and hematosis, as well as reduce breathing frequency in patients with COPD [12, 13]. These breathing techniques have been widely used clinically; however, their effects on specific patient outcomes, such as lung function, exercise capacity, dyspnea, and health-related QoL, have not been consistently identified or evaluated.

The previous related meta-analysis [7] was written over 8 years ago which mainly focused on the primary outcome of dyspnea, exercise capacity, and health-related quality of life and with the advent of over 50 newly published studies that include additional methods of interventions (IMT, EMT, and Liuzijue), with most key focus on respiratory muscle strength and endurance, also dyspnea, exercise tolerance, and patient's QoL, it is paramount to bring up to date on what we know in the treatment of patients with COPD. Thus, this review aims to summarize the results of previously published studies in evaluating the efficacy of breathing exercises in patients with COPD, as well as determine the effects of breathing exercises on respiratory muscle strength, dyspnea, exercise capacity, and health-related QoL in these patients.

## 2. Materials and Methods

This systematic review has been registered (PROSPERO registration number: CRD42018118367) and is reported according to the PRISMA guidelines [14].

### 2.1. Information Sources and Search Strategy

In order to identify relevant studies, we searched online electronic databases including PubMed, Embase, Web of Science, Cochrane Library, and Ovid until January 1, 2019. For PubMed, searches were conducted using a combination of Medical Subject Heading (MeSH) terms in the following order: chronic obstructive pulmonary disease [MeSH terms] AND breathing exercises OR respiratory muscles training. For Embase and Web of Science databases, we utilized two free-text key words: chronic obstructive lung disease and breathing exercises. For all databases, the following search filters were applied: article type (randomized controlled trials), species (humans), and language (English or any studies with English version).

### 2.2. Study Selection

Duplicate studies were excluded using Endnote X9, and subsequently, studies were screened by examining titles and abstracts that met the inclusion criteria for this review by two independent reviewers (Yun Ruisheng and Bai Yi-wen). Full-text reports of studies considered temporarily eligible and relevant were retrieved for further assessment. The second round of study evaluation was based on the following five inclusion criteria for this review. (1) Study participants were clinically diagnosed with chronic obstructive pulmonary disease using the global initiative for chronic obstructive lung disease. (2) Breathing exercises (inspiratory muscle training, expiratory muscle training, diaphragmatic exercises, and Liuzijue) were used for intervention. (3) Study designs must include randomized controlled trials (RCTs), and thus conference abstracts, pilot studies, case-control studies, expert opinions, letters, and reviews were excluded. (4) Studies must report outcome measures related to cardiopulmonary function (forced vital capacity (FVC), forced expiratory volume in 1s (FEV1), FEV1/FEV ratio (FEV1%), maximum voluntary ventilation (MVV), dyspnea from Modified Borg Scale (MBS) or any other scales, respiratory muscle strength (PImax, PEmax), and 6-minute walk test (6MWT)) and QoL (quality of life by Saint George's Respiratory Questionnaire or SF-36). (5) Studies must be published in English, or have been translated into English.

### 2.3. Assessment of Methodological Quality

The methodological quality of studies was evaluated by two reviewers initially blinded from each other's evaluation. Selection bias (random sequence generation, allocation concealment), performance bias (blinding of participants and personnel), detection bias (blinding of outcome assessments), attrition bias (incomplete outcome data), reporting bias (selective reporting), and other biases were assessed. The risk of bias was evaluated based on the following three grades: low risk, unclear risk, and high risk. Following initial evaluation, disagreements were resolved through discussion between the two reviewers or arbitrated by a third reviewer.

### 2.4. Data Items and Collection

Data regarding author information and year of publication, type of study design, sample size and characteristics of participants, method of breathing exercises, reported outcomes, and Jadad score were extracted from the studies that were selected for review, as detailed in Table 1.

### 2.5. Assessment of Evidence Synthesis

For each respiratory training technique, the evidence synthesis was based on a specific outcome. The effects of breathing exercises on lung function, including PImax, PEmax, FEV1/FVC, and Borg score, as well as 6MWT and patient QoL, were analyzed. Granted that the different specific intervention methods, ethnicity and cultures, subgroup analysis of PImax, PEmax, and patient QoL will be performed, subsequently, a cross-check was conducted by two reviewers.

Data were statistically analyzed using RevMan 5.3. Mean differences (MD) with 95% confidence intervals (95% CI) in the outcomes were estimated. Significant differences were set as *P* < 0.05. Data were synthesized using the random-effects model and given the heterogeneity of outcome measurements (*I*^2^ > 0). Statistical heterogeneity of the treatment effects among studies was assessed using Cochran's *Q* test and *I*^2^ inconsistency test; values greater than 25%, 50%, and 75% indicated low, moderate, and high heterogeneity, respectively. In order to reduce statistical heterogeneity, subgroup analysis was performed, if necessary.

## 3. Results

### 3.1. Study Selection

**Figure 1:**
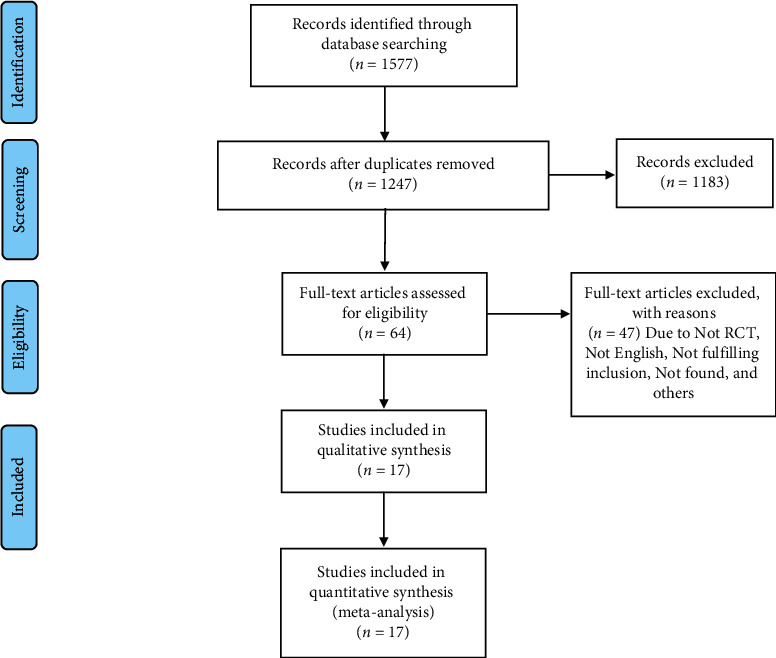
Flowchart for study selection.

A total of 1577 hits were identified from the database search, and 1247 studies remained after accounting for duplicates, as shown in Figure 1. Following reviewer screening of titles and abstracts, 64 full-text studies were assessed for eligibility. Subsequently, 47 studies were excluded for not meeting one or more of our inclusion criteria. The remaining 17 studies [3, 8, 9, 11, 15–27] were included in this review, which comprised a total of 627 participants. Detailed study search and screening process is outlined in Figure 1.

### 3.2. Characteristics of Included Studies

The 17 included trials were all published during the period from 1994 to 2019 and reported data on various outcomes related to the effects of breathing exercises on COPD patients. Of these, 14 were included in meta-analysis for PImax, 5 for PEmax, 6 for FEV1/FVC, 3 for Borg score, 10 for 6MWT, 4 for patient QoL using St. George's Respiratory Questionnaire (SGRQ), and 2 for CAT. Of note, breathing exercises are not typically used as the sole treatment technique for patients with COPD but are often combined with other treatments, such as pharmacological therapy or peripheral muscle exercise training. In addition, there are often variations in training methods among different studies, including IMT alone [3, 9, 15–20, 22–26], EMT alone [21], IMT plus EMT [8], diaphragmatic breathing [27], and Liuzijue [11]. The full summary of breathing exercise characteristics is shown in Table 1.

### 3.3. Risk of Bias

**Figure 2:**
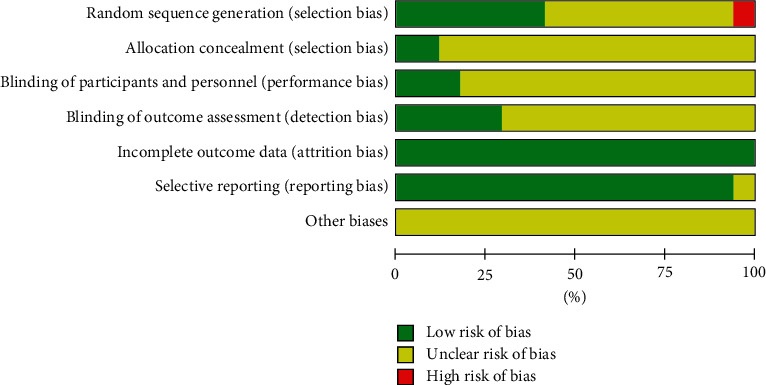
Risk of bias graph: review authors' judgement about each risk of bias item presented as percentages across all included studies.

**Figure 3:**
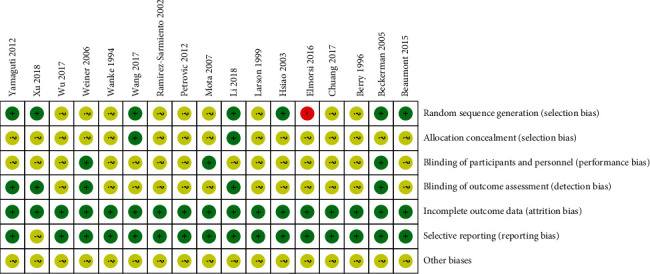
Risk of bias summary: review authors' judgements about each risk of bias item for each included study.

We further used the Cochrane Collaboration's tool for assessing the risk of bias in order to examine the methodology for all included trials. Overall, there were low risks of bias in the domains of attrition bias as well as reporting bias. In addition, there were low risks of bias in the domains of allocation concealment, blinding of participants, and personnel, as well as blinding of outcome assessment, as shown in Figure 2. The risk of bias for each individual study is illustrated in detail in Figure 3.

A total of seven trials (41.18%) reported accurate information regarding random sequence generation; two trials (11.76%) reported allocation concealment, with a low risk of selective bias; one trial (5.88%) was associated with a high risk of selective bias; and the remainder had unclear risk. Three trials (17.65%) utilized blinding of participants and personnel, with a low risk of performance bias; and the remainder had unclear risk. For detection bias, five trials (29.41%) reported the use of blinding of outcome assessment with low risk of bias; and the remainder had unclear risk. For attrition bias, all included trials had incomplete outcome data. Furthermore, 16 trials (94.12%) reported selective outcome data with low risk of bias, while the remainder had unclear risk of bias (Figure 3).

### 3.4. Effect on Strength of Respiratory Muscles

#### 3.4.1. Effects of Breathing Exercises on PImax

**Figure 4:**
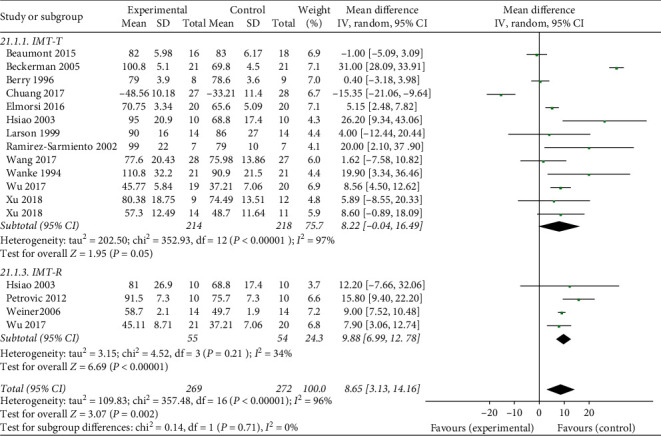
Effects of breathing exercises on P_*I*_*max*.

Of the 17 included studies, a total of 14 studies reported the effects of breathing exercises on PImax. We performed a meta-analysis with a total of 269 participants in the experimental group and 272 in the control group based on these studies, as shown in Figure 4. Subgroup analyses were performed according to the intervention, whereby the threshold IMT group showed relatively high heterogeneity (tau^2^ = 202.50, *P* < 0.01, *I*^2^ = 97%), whereas the resistance IMT group had low heterogeneity (chi^2^ = 4.52, *P*=0.21, *I*^2^ = 34%). There were also significant differences in both threshold (MD = 8.22, 95% CI: −0.04 to 16.49, *P*=0.05) and resistance (MD = 9.88, 95% CI: 6.99–12.78, *P*=0.01) between experimental and control groups in the effect of breathing exercises on PImax.

#### 3.4.2. Effects of Breathing Exercises on PEmax

**Figure 5:**
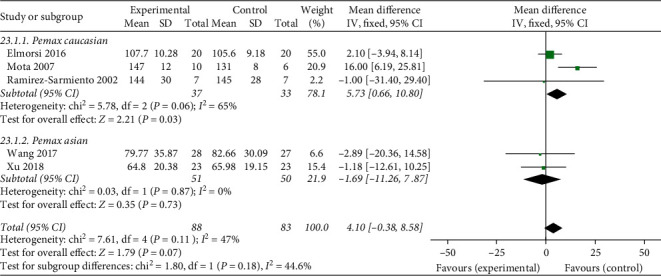
Effects of breathing exercises on P_*E*_*max*.

Of the 17 included studies, a total of 5 studies reported the effects of breathing exercises on expiratory muscle strength. We performed a meta-analysis based on these studies, which showed relatively low heterogeneity (chi^2^ = 7.61, *P*=0.11, *I*^2^ = 47%) with a total of 88 participants in the experimental group and 83 in the control group, as shown in Figure 5. Furthermore, subgroup analyses were performed according to the ethnicity, whereby the Caucasian group showed relatively moderate heterogeneity (chi^2^ = 5.78, *P*=0.03, *I*^2^ = 65%), whereas the Asian group had low heterogeneity (chi^2^ = 0.03, *P*=0.73, *I*^2^ = 0%). Although no significant differences were found between the experimental and control groups in the effect of breathing exercises on PEmax (MD = 4.10, 95% CI: −0.38 to 8.58, *P*=0.07), subgroup analysis indicates that breathing exercised could affect PEmax among Caucasians (shown in Figure 5).

### 3.5. Effect on 6MWT and QoL

#### 3.5.1. Effects of Breathing Exercises on 6-Minute Walk Test (6MWT)

**Figure 6:**
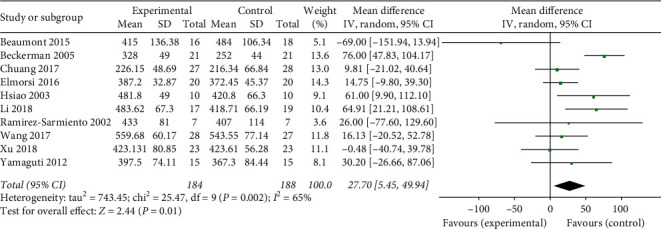
Effects of breathing exercises on 6MWT.

A total of ten studies reported the effects of breathing exercises on 6MWT. We performed a meta-analysis based on these studies, which showed relatively high heterogeneity (tau^2^ = 743.45, *P*=0.002, *I*^2^ = 65%), with a total of 184 participants in the experimental group and 188 in the control group, as shown in Figure 6. There was a significant difference between the experimental and control groups in the effect of breathing exercises on 6MWT (MD = 27.70, 95% CI: 5.45–49.94, *P*=0.01).

#### 3.5.2. Effects of Breathing Exercises on QoL

**Figure 7:**
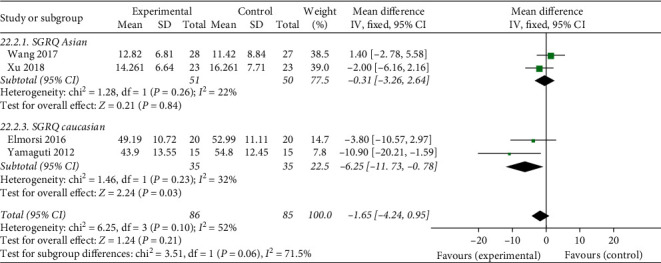
Effects of breathing exercises on QoL using SGRQ.

**Figure 8:**
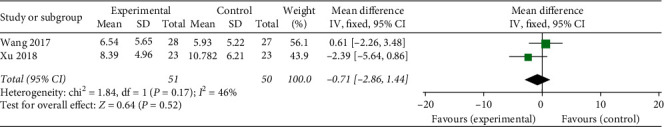
Effects of breathing exercises on QoL using CAT.

Of the 17 included studies, a total of 4 studies reported effect of breathing exercise on patient QoL, as measured using SGRQ, whereby a higher score indicated greater limitations. We performed a meta-analysis based on these studies, which showed moderate heterogeneity (tau^2^ = 8.48, *P*=0.10, *I*^2^ = 52%), with a total of 171 participants, including 86 in the experimental group and 85 in the control group, as shown in Figure 7. Results show that SGRQ scores among Caucasians have significant changes after the breathing exercise (Chi^2^ = 1.46, *P*=0.03, *I*^2^ = 32%), but no significant differences were found between the experimental and control groups in the effect of breathing exercises on patient QoL using SGRQ (MD = –1.65, 95% CI: −4.24 to 0.95, *P*=0.21). Furthermore, two studies reported patient QoL as measured using CAT, whereby a higher score indicated greater limitations in their daily lives. We performed a meta-analysis based on these studies, which showed moderate heterogeneity (chi^2^ = 1.84, *P*=0.17, *I*^2^ = 46%), with 51 participants in the experimental group and 50 in the control group, as shown in Figure 8. Likewise, no significant difference was found between the experimental and control groups in the effect of breathing exercises on patient QoL (MD = –0.71, 95% CI: −2.86 to 1.44, *P*=0.52).

## 4. Discussion

In this study, we utilized a systematic search strategy in order to identify trials from leading electronic databases. Studies were selected according to the inclusion criteria by screening titles, abstracts, and full texts, including the most recent publications. Finally, our meta-analysis included a total of 17 RCTs, comprising 627 patients with COPD according to our inclusion criteria, in order to determine the effectiveness of breathing exercises on various criteria. Our results suggested that breathing exercises were superior to control treatments for improving 6MWT and inspiratory muscle strength in patients with COPD, with both threshold and resistance training. However, breathing exercises had no significant improvement on lung function, as measured by FEV1/FVC, as well as dyspnea. Interestingly, the effects on expiratory muscle strength and patient QoL, as measured by SGRQ, showed different outcomes in a Caucasian population versus an Asian population. However, to our knowledge, no studies have examined the differences of SGRQ among different races in clinical trials or daily practice, and responsiveness of SGRQ between Caucasian and Asian. Furthermore, Lee et al. [28] suggested COPD-related comorbidities, which can be one of the reasons that affect the healthy quality of life among different races, occurred disproportionally according to race and ethnicity; thus, QoL measured by SGRQ showed different results in our paper.

The 6MWT is a test that is commonly used to reflect the walking ability and evaluation of the cardiopulmonary endurance of patients. Our results showed a significant increase in 6MWT distance in the experimental group following different kinds of breathing exercises. On the contrary, Beaumont et al. [15] showed that patients with COPD that were allowed access to an inspiratory muscle trainer with resistance actually resulted in a decrease in the 6-minute walking distance, by 14.26% (484 m).

Voluntary maximal inspiratory pressure is an important indicator of inspiratory muscle strength and lung function. Our meta-analysis results demonstrated that patients with COPD that underwent IMT had significantly improved PImax compared to those who had general training. A previous study by Gosselink et al. showed large improvements in functional exercise capacity, quality of life, and inspiratory muscle strength in patients with inspiratory muscle weakness (PImax < 60 cm H_2_O) [5]. Our present study demonstrated that patients with or without muscle weakness can improve their inspiratory muscle strength following IMT. Although the diaphragm is the most important inspiratory muscle, the intercostal, scalene, and other accessory muscles also play important roles during respiration, whereby any airflow obstruction can cause hyperinflation with incomplete expiration. Our current meta-analysis found that inspiratory resistance muscle training can result in significant improvements in inspiratory muscle strength compared with inspiratory threshold muscle training. Earlier studies had reported that IMT with or without EMT could improve inspiratory muscle strength, but there were no significant differences between groups [8]. The use of breathing exercises had been shown to provide clinical benefits in the inspiratory muscles of patients with COPD; however, the effect on expiratory muscles is unclear. Moreover, inspiratory muscle strength is typically slightly stronger than expiratory muscle strength. Our current study found no significant differences in expiratory muscle strength between experimental and control groups. However, the study by Battaglia et al. showed significant improvements in maximal expiratory pressure values after 6 to 12 months in the experimental group compared with the control group [29]. Our current meta-analysis showed that following breathing exercises, in particular EMT, expiratory muscle strength was increased in Caucasian populations but not Asian populations, which may be due to the effects of different interventions on patients of different ethnicities, and the severity of COPD.

Interestingly, our current meta-analysis showed no significant differences in the degree of airway obstruction following breathing exercises. Although pursed-lip is an effective breathing exercise for improving lung function, previous studies showed that there were no significant differences in FEV1/FVC following interventions [30]. One possible reason may be the fact that FEV1/FVC mainly reflects on the degree of obstruction, which can be difficult to ameliorate within a short timeframe using only physical therapy interventions. Moreover, the intratester and intertester reliabilities have not been formally established for FEV1/FVC. The modified Borg scale is a reliable and valid assessment tool for measuring dyspnea in patients with COPD [31]. Studies by Beaumont et al. and Petrovic et al. [15, 22] showed that inspiratory muscle strength and endurance training could improve breathing function and decrease the Borg score in patients with COPD. However, in our current meta-analysis, after analyzing the effect of breathing exercises on dyspnea using the Borg score, the results were no longer statistically significant. It is likely that patients may unconsciously and subjectively consider themselves suffering from shortness of breath even following interventions. Furthermore, assessment of lung function is not the sole criterion that can affect patient outcomes [32], and thus, stringent evaluation of other factors is key for improving the quality of life of patients with COPD.

In order to assess the effect of breathing exercises on patient QoL, our meta-analysis analyzed the SGRQ score of selected studies, which showed no significant improvements in an Asian population that were mostly comprised of mild COPD patients. Notably, there are considerable variabilities in the definitions of patient QoL and adaptability of breathing exercises across different populations [32]. However, in our current meta-analysis, a total of three studies [8, 18, 27] with a combined 61.5% weighted total for included articles reported the superiority of breathing exercises on patient QoL, including inspiratory and EMT with modified threshold trainers.

There are several limitations associated with our study. First, despite our attempt to obtain full articles for all relevant studies, there may be several studies that we missed due to them being published in other languages or mediums. Second, all studies that were included in our current meta-analysis had a relatively small sample size (<100 participants), which may result in potential bias in the validity of the results. Third, a high degree of heterogeneity was found due to patients with different severities of COPD, different outcome measure tools, or treatment durations, which may affect the accuracy of comparisons between studies. Future studies should include more large-scale RCTs that implement consistent breathing exercises and comprehensive outcome measures to better determine the effect of breathing exercises in assisting patients with COPD.

## Figures and Tables

**Table 1:** Baseline demographics, interventions, and clinical characteristics of included studies.

Author	Study design	Participants	Intervention	Outcome measures	Treatment	Jadad score
Beaumont et al. [15]	RCT	Total: 34	EG: pulmonary rehabilitation and IMT CG: pulmonary rehabilitation	(1) Lung function: P_I_max↓, Borg↓	Time: 30 min Frequency: 5 d/wk	5
		EG: 16		(2) 6MWD↓	Duration: 3 wks	
		CG: 18		(3) QoL : N/A		

Beckerman et al. [3]	RCT	Total: 42	EG: receive IMT for the next year	(1) Lung function: P_I_max↑	Time: 30 min	6
		EG: 21	CG: receive training with very low load	(2) 6MWT↑	Frequency: 6 d/wk	
		CG: 21		(3) QoL : N/A	Duration: 12 wks	

Berry et al. [16]	RCT	Total:17	EG: general exercise reconditioning and progressive threshold IMT	(1) Lung function: P_I_max -, FEV1/FVC↑, Borg-	Time: 30 min	4
		EG: 8	CG: general exercise reconditioning and sham IMT	(2) 6MWT : N/A	Frequency: 7 d/wk	
		CG:9		(3) QoL : N/A	Duration: 12 wks	

Chuang [17]	RCT	Total: 55	EG: pharmacological therapy, peripheral muscle exercise training, and IMT	(1) Lung function: P_I_max↓,	Time: 21–30 min	4
		EG: 27	CG: pharmacological therapy, peripheral muscle exercise training	(2) 6MWT↑	Frequency: 5 d/wk	
		CG: 28		(3) QoL : N/A	Duration: 8 wks	

Elmorsi [18]	RCT	Total: 40	EG: pharmacological therapy, peripheral muscle exercise training, and IMT	(1) Lung function	Time: 30 min	3
		EG: 20	CG: pharmacological therapy, peripheral muscle exercise training without IMT	P_I_max↑, P_E_max↑,	Frequency: 6 d/wk	
		CG: 20		(2) 6MWD↑ (3) QoL: (1)SGRQ-C total score↓	Duration: 8 wks	

Hsiao et al. [19]	RCT	Total: 30	EG:	(2) Lung function: P_I_max↑	Time: 30 min	5
		EG:	TG: receiving pressure threshold IMT	(3) 6MWT↑	Frequency: 5 d/wk	
		TG:10	TRG: receiving targeted resistive IMT	(4) QoL : N/A	Duration: 8 wks	
		TRG: 10	CG: no IMT			
		CG: 10				

Larson et al. [20]	RCT	Total: 28	EG: trained as described above	(1) Lung function: P_I_max↑	Time: 30 min	4
		EG: 14	CG: performed at home on a calibrated stationary cycle ergometer	(2) 6MWT : N/A	Frequency: 5 d/wk	
		CG: 14		(3) QoL : N/A	Duration: 16 wk	

Li et al. [11]	RCT	Total: 36	EG: Liuzijue exercise according to the health Qigong Liuzijue program	(1) Lung function: FEV1/FVC↑	Time: 60 min	6
		EG: 17	CG: pharmacological therapy, smoking cession, and education without any exercise interventions	(2) 6MWT↑	Frequency: 6 d/wk	
		CG: 19		(3) QoL : N/A	Duration: 24 wks	

Mota et al. [21]	RCT	Total: 16	EG: equivalent to around 50% of their maximal expiratory threshold device	(1) Lung function: P_E_max↑,	Time: 30 min	5
		EG: 10	CG: no additional loads	(2) FEV1/FVC↓	Frequency: 3 d/wk	
		CG: 6		(3) 6MWT : N/A QoL : N/A	Duration: 5 wks	

Petrovic et al. [22]	RCT	Total: 20	EG: inspiratory muscle strength and endurance training	(1) Lung function: P_I_max↑, Borg scale↓	Time: 15 min	4
		EG: 10	CG: without inspiratory muscle training	(2) 6MWT : N/A	Frequency: 7 d/wk	
		CG: 10		(3) QoL : N/A	Duration: 8 wks	

Ramrez-sarmiento et al. [23]	RCT	Total: 14	EG: through IMT device	(1) Lung function:	Time: 30 min	4
		EG: 7	CG: through the same IMT device with no additional load	(2) P_I_max↑, P_E_max↓	Frequency: 5 d/wk	
		CG: 7		(3) 6MWT↑ QoL : N/A	Duration: 5 wks	

Wang et al. [9]	RCT	Total: 55	EG: 8 weeks of CET + IMT	(1) Lung function: P_I_max↑, P_E_max↓, FEV1/FVC↑,	Time: 30 min	5
		EG: 28	CG: 8 eeks of CET alone	(2) 6MWT↑	Frequency: 3 d/wk	
		CG: 27		(3) QoL : SGRQ, CAT	Duration: 8 wks	

Wankle et al. [24]	RCT	Total: 42	EG: pharmacological therapy, peripheral muscle exercise training	(1) Lung function: P_I_max↑	Time: 30 min	4
		EG: 21	CG: pharmacological therapy, peripheral muscle exercise training, and IMT	(2) 6MWT : N/A	Frequency: 7 d/wk	
		CG: 21		(3) QoL : N/A	Duration: 8 wks	

Weiner and Weiner [25]	RCT	Total: 28	EG: pharmacological treatment with low PIF SIMT	(1) Lung function: P_I_max↑	Time: 60 min	5
		EG: 14	CG: only pharmacological treatment	(2) 6MWT-	Frequency: 6 d/wk	
		CG: 14		(3) QoL : N/A	Duration: 8 wks	

Wu et al. [26]	RCT	Total: 60	EG:	(1) Lung function: P_I_max↑, FEV1/FVC↓, dyspnea	Time: 30 min	4
		EG:	R-IMT group: using resistive load devices, and IMT	(2) 6MWT : N/A	Frequency: 7 d/wk	
		R-IMT: 21,	T-IMT group: using threshold load devices		Duration: 8 wks	
		T-IMT: 19,	CG: only with pharmacological therapy without any kind of pulmonary rehabilitation			
		CG: 20				

Xu [8]	RCT	Total: 46	EG: a half of IMT and a half of no-load respiratory muscle training	(1) Lung function: P_I_max↑, P_E_max↓, FEV1/FVC	Time: 48 min	4
		EG: 23	CG: no-load respiratory muscle training daily	(2) 6MWT-	Frequency: 7 d/wk	
		CG: 23		(3) QoL : CAT, SGRQ↓	Duration: 8 wks	

Yamaguti et al. [27]	RCT	Total: 30	EG: diaphragmatic breathing training program	(1) Lung function: FEV1/FVC↓	Time: 45 min	5
		EG: 15	CG: usual care	(2) 6MWT↑	Frequency: 3 d/wk	
		CG: 15		(3) QoL : SGRQ↓	Duration: 4 wks	

RCT, randomized controlled trial; EG, experimental group; CG, control group; IMT, inspiratory muscle training; N/A, not available; SEMT, specific expiratory muscle training; SIMT, specific inspiratory muscle training; TG, threshold group; TRG, targeted resistive group; wk, week.
